# Editorial: Local and traditional medicine in regulation of the cancer immune suppression microenvironment, Volume II

**DOI:** 10.3389/fphar.2023.1192478

**Published:** 2023-05-12

**Authors:** Shicui Hong, Neng Wang, Zhiyu Wang

**Affiliations:** ^1^ The Second Clinical College of Guangzhou University of Chinese Medicine, Guangdong Provincial Hospital of Chinese Medicine, Guangzhou, China; ^2^ State Key Laboratory of Dampness Syndrome of Chinese Medicine, The Second Affiliated Hospital of Guangzhou University of Chinese Medicine, Guangzhou, China; ^3^ The Research Center for Integrative Medicine, School of Basic Medical Sciences, Guangzhou University of Chinese Medicine, Guangzhou, Guangdong, China; ^4^ Guangdong-Hong Kong-Macau Joint Lab on Chinese Medicine and Immune Disease Research, Guangzhou University of Chinese Medicine, Guangzhou, Guangdong, China; ^5^ Guangdong Provincial Key Laboratory of Clinical Research on Traditional Chinese Medicine Syndrome, Guangdong Provincial Academy of Chinese Medical Sciences, Guangdong Provincial Hospital of Chinese Medicine, Guangzhou, Guangdong, China

**Keywords:** local and traditional medicine, immunotherapy, tumor microenvironment, cancer, omics

## Introduction

Avoiding immune destruction has been considered as one of cancer hallmarks in 2011 (Hanahan and Weinberg, 2011). The recruitment of M2 macrophages, regulatory T cells (Tregs), and myeloid-derived suppressor cells leads to immune suppression and the recurrence and metastasis of cancer, and the dysregulation of immune checkpoints further aggravates the escape of cancer cells from the cytotoxic killing strategies (He and Xu, 2020). In recent decades, immunotherapies have been paid increasing attention worldwide due to their exciting clinical efficacies in melanoma, acute myeloid leukemia, and lung cancer, *etc.* However, only around 20% of cancer patients are responsive to immunotherapy (Sharma et al., 2017), most malignancies are termed as “cold tumor” due to their low infiltration of T cells (Bonaventura et al., 2019). Meanwhile, systemic adverse effects and drug resistance also significantly limit the clinical application of immuno-targeting strategies (Murciano-Goroff et al., 2020). It is necessary and urgent to develop a cocktail treatment strategy to activate the tumor immunogenicity with enhanced efficacy and safety.

Local and traditional medicine has long been applied for cancer adjuvant prevention and treatment due to its holistic regulation on multi-targets, multi-metabolites and multi-mechanisms, especially in Asian counties. A number of formulas have been demonstrated effective in modulating the tumor immune microenvironment. For example, XIAOPI formula was found to inhibit breast cancer growth and metastasis via suppressing macrophage M2 polarization and CXCL1 expression (Zheng et al., 2020). Aiduqing formula was also validated effective in inhibiting Treg differentiation and recruitment in breast cancer (Li et al., 2021). Meanwhile, numerous clinical trials also proved that traditional medicine has significant priorities in improving cancer patients’ survival period and quality of life, which is closely associated with the normalization of tumor immune microenvironment (Zhang et al., 2021). Nevertheless, the “herb-ingredient-target” regulation network underlying the immune regulation effects of local and traditional medicine is still awaiting to be explored, and corresponding technologies are also required to be developed to reveal the mystery mask of the complicated regulation network. Focusing on this research topic, a total of four manuscripts have been accepted for publication, of which one is original research and three are reviews.

Bojungikki-Tang (BJIKT), known as Bu-Zhong-Yi-Qi-Tang in China, has been reported to augment the anti-cancer immune activities by enhancing natural killer cell activity and restoring cytotoxic T cell response in both *in vitro* and *in vivo* models (Li et al., 1999) (Liu et al., 2021). However, its synergistic effects with immune checkpoint inhibitors are remained unclear. Chun et al.found that the combined BJIKT and anti-PD-L1 therapy could suppress tumor growth and increase cytotoxic T lymphocytes and natural killer cells in the MC38 colon cancer-bearing mouse model. Interestingly, BJIKT exhibited little cytotoxic effects on colon cancer cells directly. Moreover, BJIKT was found to suppress myeloid-derived suppressor cells, and therefore contributing to the increased tumor infiltrating T lymphocytes. The results highlight the potential role of traditional medicine as an adjutant therapy enhancing the anti-cancer immune activities when combined with immune checkpoint inhibitors.

The infiltration of immune suppression cells into tumor tissues is closely correlated with pro-angiogenic factors. Anti-angiogenesis therapy has been widely accepted as a cancer cell killing strategy, but always challenged by drug resistance(Neves et al., 2020). Nowadays the combination of anti-angiogenesis and immune therapy has been considered as a promising strategy in solid cancer treatment. Interestingly, Zhou et al.suggested that local and traditional medicine should be the easier track to identify the candidate inhibitor co-targeting angiogenesis and immune microenvironment. A number of phytochemicals, such as ginseng, silybin, luteolin and bufalin, have been demonstrated with dual targeting properties. Meanwhile, the development of nanosystem delivery technology make it a reality co-targeting neoangiogenesis and immune cells by loading two or more phytochemicals. More research studies are expecting to investigate the crosstalk between angiogenesis and immune microenvironment remodeling from the perspective of botanical medicine.

Premetastatic niche formation (PMN) was firstly discovered by (Kaplan et al., 2005). It is hypothesized that the establishment of immune-suppression microenvironment is a prerequisite for cancer cell seeding. In other words, the site of immune-suppression microenvironment determines the site of metastasis. Prostate cancer has a tendency towards bone metastasis, Chen et al. presented a new concept called premetastatic bone niche, which claimed that the balance disruption between osteoblast and osteoclast would firstly mobilize metastasis-initiating cancer cells seeding, and subsequently remodeled extracellular matrix to recruit hematopoietic progenitor cells facilitating the maturation of PMN. Fortunately, based on the Yin-Yang theory, accumulating evidence found that traditional medicine can suppress PMN formation in breast cancer, gastric cancer and colorectal cancer. Meanwhile, a lot of active compounds derived from herbs also showed the ability of regulating PMN, such as celastrol, Perillaldehyde, and bufalin, *etc.* However, further in-depth studies are also needed to illuminate the network regulation of PMN formation, as well as the network pharmacology of traditional medicine.

With the development of OMICs technologies, the exploration of “herb-ingredient-target” network regulation of local and traditional medicine has shifted from black box to white box. The Chinese medicinal tonics are considered as effective adjuvants against gastrointestinal cancer. Zuo et al. reviewed OMICs-based research progress of tonics on gastrointestinal malignancies from the perspectives of proteomics, transcriptomics, genomics and metabolomics. The results showed that the targets of tonics included sustaining proliferative signaling, resisting cell death, activating invasion and metastasis, inducing angiogenesis, deregulating cellular energetics, inflammation-mediated carcinogenesis, and genomic instability and mutation. Based on the findings, OMICS presented priorities of small sample size, large-scale and high-throughput screening. However, the comprehensive analysis algorithm between OMICS data is still awaiting to be developed, and more attention should be paid to glycomics and lipomics technologies.

Overall, these publications in this Research Topic are expected to advance our understanding of local and traditional medicine in cancer immune microenvironment regulation. Based on thousands of years of application and current advanced technologies, traditional medicine not only has become a fast avenue to discover novel drugs for cancer prevention and treatment, but also shows its scientific connotation when incorporated with chemotherapy, radiotherapy or immunotherapy. Future studies are expected to validate the regulatory effects of traditional medicine on cancer immune microenvironment with advanced molecular target fishing technologies, comprehensive OMICS analysis, and large-scale clinical studies. Moreover, it is worthwhile to reach an international consensus on the application of traditional medicine in preventing and treating malignancies.
